# Changes in Vertebral Bone Density and Paraspinal Muscle Morphology Following Spaceflight and 1 Year Readaptation on Earth

**DOI:** 10.1002/jbm4.10810

**Published:** 2023-11-08

**Authors:** Jennifer C. Coulombe, Fjola Johannesdottir, Katelyn A. Burkhart, Henriette Brummer, Brett T. Allaire, Mary L. Bouxsein

**Affiliations:** ^1^ Center for Advanced Orthopedic Studies, Department of Orthopedic Surgery Beth Israel Deaconess Medical Center Boston Massachusetts USA; ^2^ Department of Orthopaedic Surgery Harvard Medical School Boston Massachusetts USA

**Keywords:** ANALYSIS/QUANTITATION OF BONE, BONE QCT/MICROCT, BONE‐MUSCLE INTERACTIONS, DISEASES AND DISORDERS OF/RELATED TO BONE, OTHER, SYSTEMS BIOLOGY‐BONE INTERACTORS

## Abstract

Astronauts have an increased risk of back pain and disc herniation upon returning to Earth. Thus, it is imperative to understand the effects of spaceflight and readaptation to gravity on the musculoskeletal tissues of the spine. Here we investigated whether ~6 months of spaceflight led to regional differences in bone loss within the vertebral body. Additionally, we evaluated the relationships between vertebral bone density and paraspinal muscle morphology before flight, after flight, and after readaptation on Earth. We measured vertebral trabecular bone mineral density (Tb.BMD), paraspinal muscle cross‐sectional area (CSA), and muscle density in 17 astronauts using computed tomography (CT) images of the lumbar spine obtained before flight (before flight, *n* = 17), after flight (spaceflight, *n* = 17), and ~12 months of readaptation to gravitational loading on Earth (follow‐up, *n* = 15). Spaceflight‐induced declines in Tb.BMD were greater in the superior region of the vertebral body (−6.7%) than the inferior (−3.1%, *p* = 0.052 versus superior region) and transverse regions (−4.3%, *p* = 0.057 versus superior region). After a year of readaptation to Earth's gravity, Tb.BMD in the transverse region remained significantly below preflight levels (−4.66%, *p* = 0.0094). Paraspinal muscle CSA and muscle density declined −1.0% (*p* = 0.005) and −0.83% (*p* = 0.001) per month of spaceflight, respectively. Ultimately, bone loss in the superior vertebral body, along with fatty infiltration of paraspinal muscles and incomplete recovery even after a year of readaptation on Earth, may contribute to spinal pathology in long‐duration astronauts. © 2023 The Authors. *JBMR Plus* published by Wiley Periodicals LLC on behalf of American Society for Bone and Mineral Research.

## Graphical abstract

Astronauts have an increased risk of back pain and disc herniation upon returning to Earth, so it is imperative to understand the effects of spaceflight and readaptation to gravity on the musculoskeletal tissues of the spine. Spaceflight‐induced declines in astronauts’ vertebral Tb.BMD were greater in the superior region of the vertebral body (−6.7%) than the inferior (−3.1%, *p* = 0.052 versus superior region) and transverse regions (−4.3%, *p* = 0.057 versus superior region). After a year of readaptation to Earth's gravity, Tb.BMD in the transverse regions remained significantly below preflight levels (−4.66%, *p* = 0.0094). Bone loss in the transverse vertebral body and incomplete recovery even after a year of readaptation on Earth may contribute to spinal pathology in long‐duration astronauts.

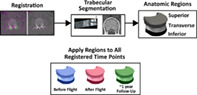

## Introduction

Musculoskeletal decrements, particularly the potential for early onset osteoporosis and elevated risk of fracture, back pain, and disc herniation, due to long‐duration spaceflight are a major concern for astronauts upon return to Earth. Previous studies reported bone mineral density (BMD) losses of 1% to 2%/month at the spine during spaceflight, with incomplete recovery for some even 2 years after a mission.^(^ ^1^ ^)^ About 40% of astronauts suffer from low back pain upon return to Earth.^(^ ^2^, ^3^ ^)^ Astronauts also experience an elevated risk of disc injury following their return to Earth. Specifically, 10% of US astronauts experience a disc herniation upon their return, most within the first year, a rate that is 4.3 times greater than the general population.^(^ ^4^ ^)^

The mechanisms underlying the relatively high incidence of back pain and disc herniation in astronauts have yet to be elucidated but may include damage to the vertebral endplates, changes in intravertebral bone density distribution, and/or altered paraspinal muscle morphology. Vertebral endplates are thin, cartilaginous layers between the vertebral bodies and the intervertebral disc that act to transfer stresses between the vertebral body and the intervertebral disc and to transport nutrients between the disc cells and vertebral capillaries.^(^ ^5^ ^)^ Damage to the vertebral endplates has been associated with both disc degeneration and vertebral fractures in aging populations.^(^ ^6^ ^)^ Maintaining vertebral bone density is crucial for preventing the degradation of endplates.

Variations in the spatial distribution of trabecular bone density within the vertebral body are associated with age‐related vertebral fractures.^(^ ^7^ ^)^ Like aging, spaceflight may alter the distribution of trabecular bone within the vertebral body. In particular, loss of bone density in the superior and inferior regions of the vertebral body may be indicative of endplate degradation and/or lead to altered endplate mechanics. However, previous studies of the effects of spaceflight on vertebral bone health relied upon integrative measures of spinal bone density derived either from dual‐energy X‐ray absorptiometry^(^ ^8^ ^)^ or computed tomography (CT) scans.^(^ ^9^ ^)^ Therefore, it remains unknown whether the distribution of vertebral trabecular bone density is altered due to spaceflight and whether changes in the distribution of bone density within the vertebral body persist following the return to Earth.

Previous studies hypothesized that disc volume changes (i.e., hydration and swelling) due to unloading increased the risk of herniation^(^ ^4^, ^10^, ^11^, ^12^ ^)^; however, others found the disc volume change negligible or transient in astronauts.^(^ ^3^ ^)^ Instead, the pathophysiology of postflight lower back pain and disc pathology may be due to deficits in paraspinal musculature.^(^ ^13^ ^)^ In terrestrial patients, low back pain is associated with muscle atrophy and fatty infiltration of the lumbar paraspinal muscles.^(^ ^14^ ^)^ The degradation of these key spine‐stabilizing muscles may result in altered posture, biomechanical loading patterns, and spinal stiffening.^(^ ^15^ ^)^ Spaceflight leads to loss of trunk muscle size and increased fatty infiltration, with inconsistent reports on the rate and extent of recovery following return to Earth.^(^ ^9^, ^16^ ^)^ Yet, whether these changes in paraspinal muscles are associated with losses of vertebral bone density has not been investigated. Changes in the relationships between paraspinal muscle density, size, and vertebral bone density may reflect altered loading patterns in the spine that contribute to spinal pathologies in astronauts.

Thus, this study aimed to use CT scans of the lumbar spine from long‐duration astronauts to determine the effect of spaceflight and 12 months of reloading on the spatial heterogeneity of vertebral trabecular bone density. We hypothesized that long‐duration spaceflight disproportionately affected the superior and inferior regions of the vertebral body and might contribute to elevated rates of endplate pathologies and disc herniation. Additionally, we investigated the relationship between vertebral bone density and paraspinal muscle cross‐sectional area (CSA) and density before and after long‐duration spaceflight, hypothesizing that long‐duration spaceflight altered the relationship between paraspinal muscle morphology and vertebral bone density, which could contribute to the elevated risk of disc degeneration and back pain in astronauts.

## Materials and Methods

We leveraged previously collected three‐dimensional (3D) CT scans of the lumbar spine from the NASA CT study of bone health.^(^ ^1^, ^9^ ^)^ In total, 17 crewmembers were enrolled, including nine astronauts and eight cosmonauts. Within this group of astronauts and cosmonauts, two were in space for 4 months, three for 5 months, seven for 6 months, and five for 7 months. The Institutional Review Boards approved the study of NASA's Johnson Space Center and Beth Israel Deaconess Medical Center, and all participants provided written informed consent.

### Lumbar CT image acquisition

We used previously collected 3D CT scans (GE HIspeed Advantage, GE Medical Systems, Milwaukee, WI, USA) of the lumbar spine that were acquired at three time points^(^ ^17^ ^)^: before flight (*n* = 17), immediately after flight (*n* = 17), and ~12 months readaptation to gravitational loading on Earth (follow‐up, *n* = 15). Preflight CT scans were acquired 30–60 days before the mission, and postflight CT scans occurred 7–10 days after the return to Earth. We evaluated CT images (80 kVp, 280 mA, 3‐mm‐thick slices) of the first lumbar vertebrae (L1). A hydroxyapatite phantom (QCT‐Bone Mineral™ phantom; Image Analysis, Inc., Columbia, KY, USA) was simultaneously scanned with each subject for the conversion of CT Hounsfield units (HU) to BMD (in milligrams per cubic centimeter [mg/cm^3^]" hydroxyapatite).

**Fig. 1:**
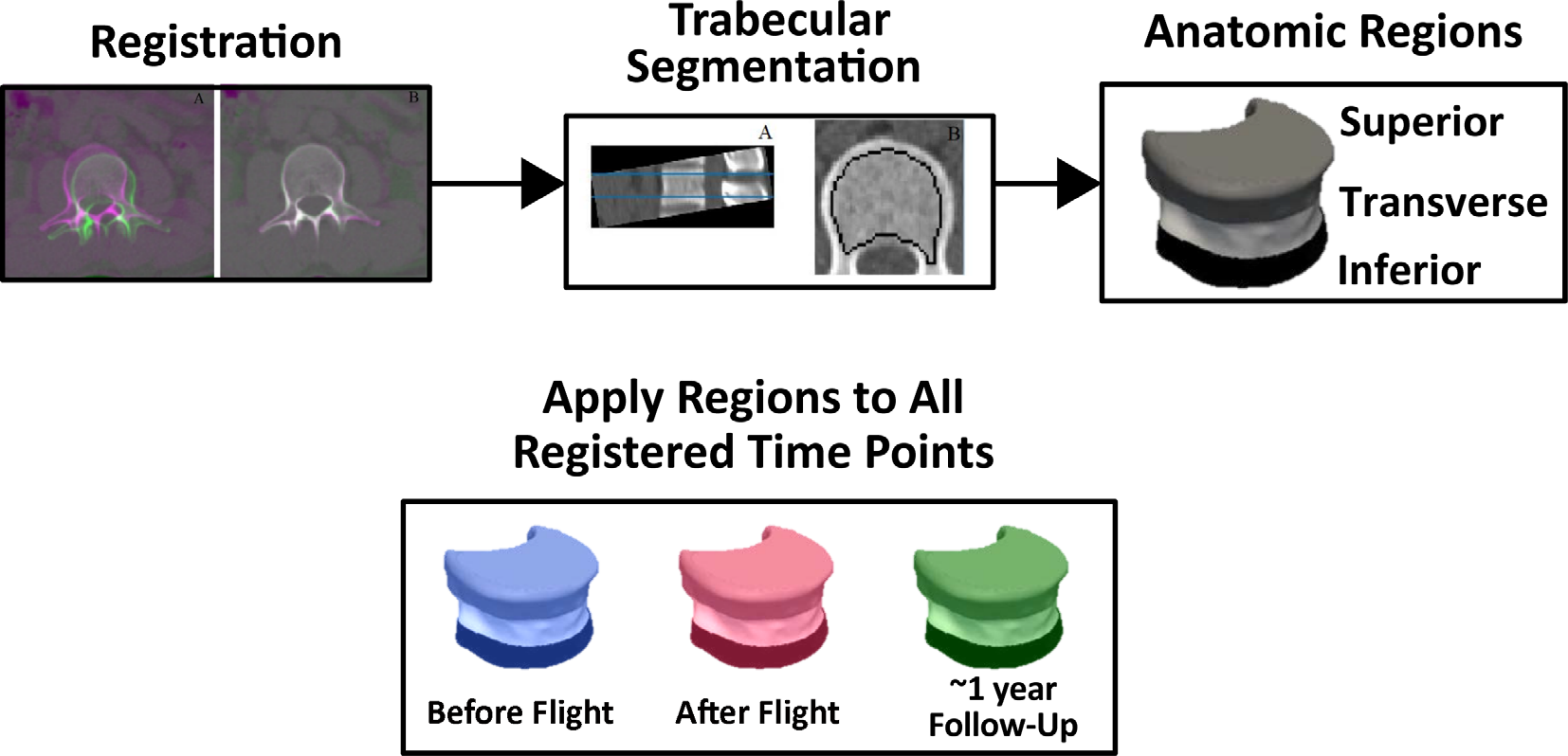
The L1 vertebral bodies from postflight CT scans were registered to preflight images and trabecular BMD was measured in the superior, transverse, and inferior regions of the vertebral body.

As previously reported, prior to bone assessment, postflight and follow‐up scans were registered to preflight scans using rigid registration in MATLAB R2017a (The MathWorks Inc., Natick, MA, USA).^(^ ^9^ ^)^ Next, we evaluated the spatial heterogeneity of vertebral bone density using a semiautomatic segmentation protocol, whereby the three regions within the vertebral body^(^ ^7^ ^)^ were extracted from preflight scans (Fig. 1). Briefly, we excluded cortical bone and endplates and created a trabecular bone mask, which was further automatically divided into three anatomical regions (inferior, superior, and transverse). We used an automatic mapping algorithm (MATLAB R2016b, The MathWorks Inc., Natick, MA, USA) to register the trabecular bone masks extracted from preflight to postflight and follow‐up scans. BMD from the entire trabecular mask was defined as global trabecular BMD (global Tb.vBMD). Finally, we calculated BMD from each anatomical region (regional bone mineral density, rBMD, mg/cm^3^) from these matched regions of interest.

### CT‐based measurements of paraspinal muscles at L1

The CSAs (mm^2^) of the paraspinal muscles were measured at the L1 mid‐vertebral body according to previously published methods.^(^ ^9^ ^)^ For this analysis, we summed the areas of the paraspinal muscles (erector spinae, transversospinalis, psoas major, and quadratus lumborum) to compute a total paraspinal muscle area. Muscle density was taken as the mean of voxel attenuation in HU within each muscle, averaging the right and left sides. Voxels outside the range of −50 to 150 HU were excluded before CSAs and density were calculated to remove voxels of pure fat, tendon, and bone along the periphery of the muscle contours. Total paraspinal muscle density was calculated as a weighted average by individual muscle size. Muscle measurements were performed by a single operator, with intraclass correlation coefficients of >0.75 for 95% of all muscle CSAs.^(^ ^9^ ^)^

### Statistical analyses

Standard descriptive statistics of global (Tb.BMD; total trabecular BMD of the vertebral body) and regional trabecular BMD (rBMD; superior, transverse, inferior), total paraspinal muscle CSA, and total paraspinal muscle density are presented as mean ± SD. As mission duration varied by crewmember, we also computed each variable's monthly rate of change by dividing the percentage difference between postflight and preflight values by mission duration. Mission duration was not a significant predictor of vBMD or rBMD. Differences between time points were evaluated via paired *t*‐tests. We used a one‐sample *t*‐test to determine whether the monthly rate of change of these variables was significantly different from 0. Next, we evaluated the associations among global Tb.BMD, paraspinal muscle density, and CSA via general linear regression models. Lastly, to evaluate whether spaceflight disproportionately affected specific regions of the vertebral body, we computed the monthly rate of change for each of the three regions for spaceflight and readaptation as described earlier. Statistical analyses were performed with R version 4.0.0 (R Foundation for Statistical Computing, Vienna, Austria). *p* values ≤0.05 were considered statistically significant.

## Results

### Study sample

The astronauts enrolled in this study had a mean (±SD) age of 45 ± 4 years, height of 173.5 ± 5.6 cm, weight of 76.5 ± 7.1 kg, and mission duration of 5.9 ± 1.0 months.

### Vertebral trabecular BMD loss following spaceflight is spatially dependent

**Fig. 2:**
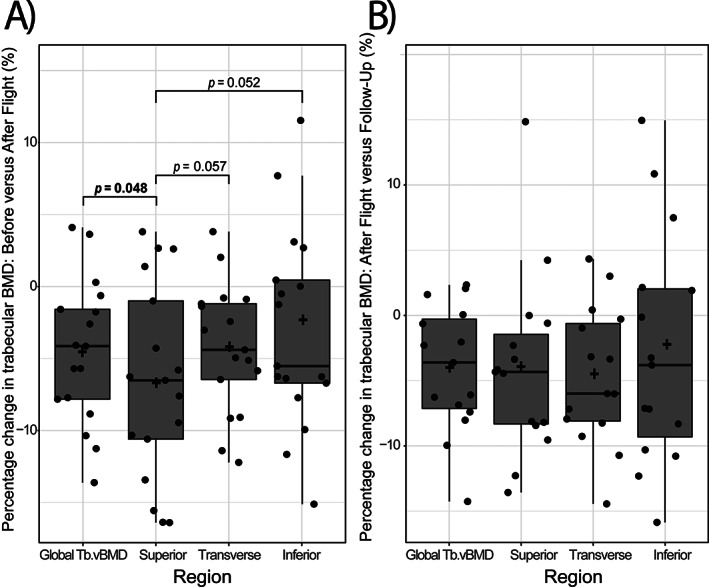
Percentage changes in global and regional trabecular (BMD) versus before flight following (*A*) long‐duration spaceflight and (*B*) 1 year readaptation on Earth. Data are presented as a box‐and‐whisker plot where each point represents the percentage change of a single astronaut, with the plus sign representing the mean percentage change per region.

The magnitude of trabecular bone loss in the superior region of the vertebral body (−6.7%) was greater than in the inferior (−3.1%, *p* = 0.052 versus superior region) or the transverse (−4.3%, *p* = 0.057 versus superior region) regions (Fig. 2A, Table 1). Trabecular bone loss following spaceflight was significantly greater in the superior region than for average trabecular BMD of the entire vertebral body (*p* = 0.0481).

**Table 1:** Regional Trabecular BMD (rBMD) Values in L1 Vertebral Body of ISS Crewmembers before Flight, after Flight, and at Follow‐Up after 1 Year Readaptation on Earth

	Preflight values (*n* = 17) mg/cm^3^	Postflight percentage difference from preflight value (*n* = 17)	*p* value (difference from preflight value)	Follow‐up percentage difference from preflight value (*n* = 15)	*p* value (difference from preflight value)
Global Tb.vBMD	182.3 ± 29.5	−4.57% ± 5.02%	***p* = 0.002**	−4.09% ± 4.85%	*p* = 0.37
Superior rBMD	178.9 ± 32.4	−6.65% ± 6.79%*	***p* = 0.0013**	−4.02% ± 7.07%*	*p* = 0.38
Transverse rBMD	177.5 ± 29.6	−4.26% ± 4.48%*	***p* = 0.0017**	−4.66% ± 5.28%*	*p* = 0.0094
Inferior rBMD	196.5 ± 32.4	−3.05% ± 6.94%	***p = 0.0598***	−2.78% ± 8.91%	*p* = 0.23

*Note*: Bold values indicate any *p* values that are significant by *p* < 0.05. Italic values indicate any *p* valuse that are a trend 0.75 < *p* < 0.05.

We next examined whether regions within the vertebra exhibited differential readaptation to gravitational loading. After 1 year on Earth, rBMD in the inferior region and the superior region did not differ from preflight values (*p* = 0.23; *p* = 0.38). Yet, in the transverse region, follow‐up trabecular rBMD values remained significantly less −4.7% than preflight values *p* = 0.009) (Table 1).

**Fig. 3:**
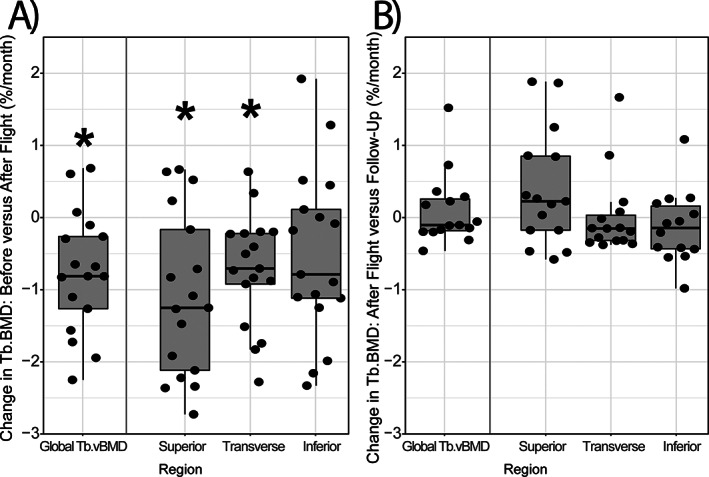
Monthly percentage changes in global and regional trabecular BMD during (*A*) long‐duration spaceflight mission and (*B*) in 1 year readaptation to gravitational loading on Earth. The rate of change in trabecular bone density during spaceflight was calculated as the percentage difference between preflight and immediate postflight measures, divided by individual astronauts' mission duration in months. The rate of change in trabecular bone density during readaptation was calculated as the percentage difference in values at postflight and 1‐year follow‐up time points, divided by individual astronauts' mission duration in months. Each data point represents the monthly percentage change of a single astronaut.

Given the regional variation in trabecular bone loss within the L1 vertebral body during a mission and readaptation, we then examined the monthly rates of change within the different regions of L1 vertebrae to account for variations in mission duration. In the superior region, trabecular bone density declined −1.08%/month during spaceflight (*p* = 0.0012, Fig. 3 *A*, Table 2), with a recovery rate of +0.39%/month during readaptation on Earth (*p* = 0.0790, Fig. 3 *B*, Table 2). In comparison, trabecular bone density in the transverse region exhibited a −0.72%/month decline during spaceflight (*p* = 0.0015, Fig. 3 *A*, Table 2), with no change during the 1‐year readaptation period (+0.01%/month, *p* = 0.9373, Fig. 3B, Table 2). Unlike the superior and transverse regions, trabecular bone density in the inferior region was unchanged during both spaceflight (−0.51%/month, *p* = 0.94) and readaptation (+0.05%/month, *p* = 0.82, Table 2).

**Table 2:** Changes in Trabecular BMD in Different Regions of L1 Vertebral Body Due to Spaceflight and After 1 Year Readaptation on Earth (mean ± SD)

Vertebral region	Change from preflight value (% per month)	*p* value (difference from 0)	Change from postflight value (% per month)	*p* value (difference from 0)
Total vertebral trabecular	−0.76 ± 83	***p* = 0.002**	0.10 ± 50	*p* = 0.434
Superior	−1.08 ± 1.13	***p* = 0.0012**	0.39 ± 0.80	*p* = 0.079
Transverse	−0.72 ± 0.77	***p* = 0.0015**	0.01 ± 0.56	*p* = 0.94
Inferior	−0.51 ± 1.17	*p* = 0.093	0.05 ± 0.85	*p* = 0.82

*Note*: Bold values indicate any *p* values that are significant by *p* < 0.05. Italic values indicate any *p* valuse that are a trend 0.75 < *p* < 0.05.

### Changes in paraspinal muscle morphology

Total paraspinal muscle cross‐sectional area and total muscle density at L1 of astronauts and cosmonauts were evaluated in this study. Spaceflight led to an average (±SD) −1.02% ± 1.29%/month decline of paraspinal muscle CSA (*p* = 0.005) and −0.83% ± 0.89%/month decline of paraspinal muscle density (*p* = 0.001). During the year following return to Earth, there was a trend toward recovery of paraspinal muscle density at a rate of 0.28% ± 0.55%/month (*p* = 0.067). In comparison, paraspinal muscle CSA increased significantly from postflight levels at a rate of +0.72% ± 0.78%/month (*p* = 0.0032) during a year of readaptation to loading on Earth.

### Heterogeneity in spinal muscle and bone changes with spaceflight and readaptation to gravitational loading

**Fig. 4:**
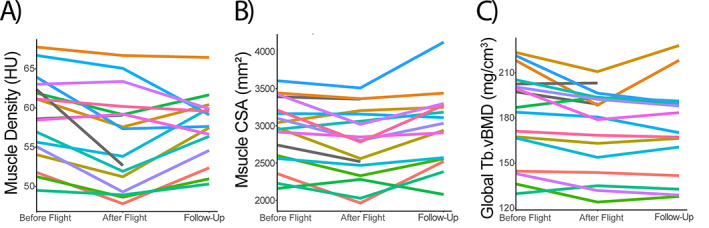
Changes in paraspinal muscle morphology and lumbar vertebral bone density are highly variable in astronaut population following long‐duration spaceflight and 1 year of readaptation on Earth. Spaghetti plots of (*A*) paraspinal muscle density, (*B*) paraspinal muscle cross‐sectional area, and (*C*) vertebral trabecular BMD before flight, after flight, and at follow‐up (~12 months readaptation on Earth). Each astronaut is represented by their own line. Two astronauts did not complete the follow‐up measure (gray lines).

Bone and muscle changes in the spine following both spaceflight mission and readaptation period varied among individuals. Per month of spaceflight, astronauts experienced an average −0.76% decline of Global Tb.vBMD (*p* = 0.002), −1.02% decline of muscle CSA (*p* = 0.005), and −0.83% decline of muscle density (*p* = 0.001). However, these changes were not uniform among crewmembers (Fig. 4). For example, some astronauts experienced limited changes in muscle density (i.e., dark orange and light green line, Fig. 4*A*) or bone density (i.e., light red line, purple line, tan line, Fig. 4*C*) due to spaceflight. Similarly, there was variability in the readaptation of bone and muscle density and muscle CSA. During the readaptation period, the year following their return to Earth, on average global Tb.vBMD was regained at a nonsignificant average rate of +0.10%/month (*p* = 0.434). Yet some astronauts did return to preflight global Tb.vBMD levels (i.e., light and dark orange lines, Fig. 4*C*). Similarly, there was a trend toward increased muscle density at a rate of 0.28%/month (*p* = 0.067), despite several astronauts having lower than preflight muscle density measures a year after their return to Earth (i.e., light purple and light blue lines, Fig. 4*A*). By contrast, muscle CSA increased from postflight levels at a rate of +0.72%/month (*p* = 0.0032) during a year of readaptation to loading on Earth.

### Relationship between trunk muscle and vertebral bone density with spaceflight and readaptation to gravitational loading

**Fig. 5:**
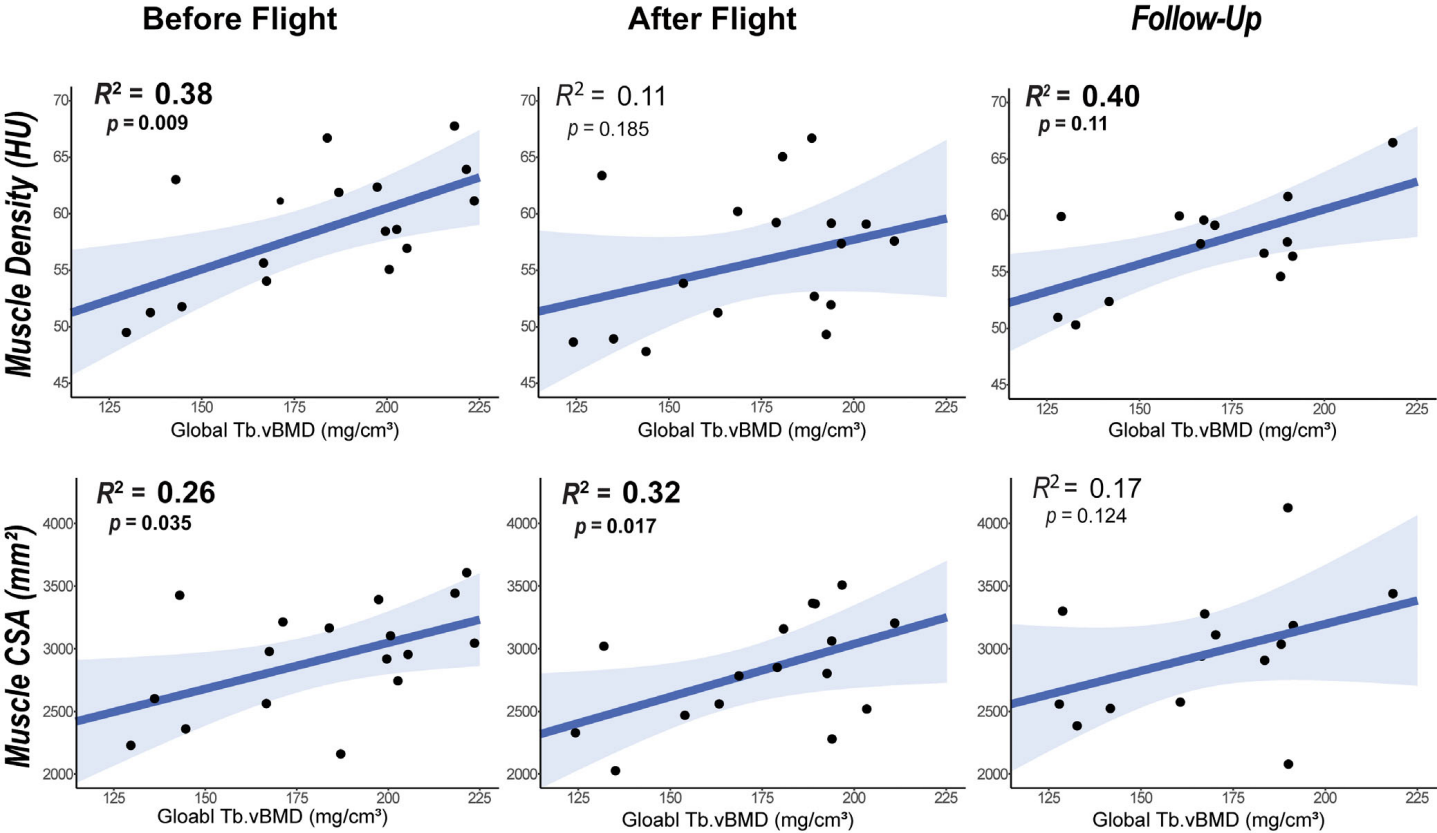
Relationships between global trabecular BMD (Tb.vBMD) and paraspinal muscle metrics before flight, after flight, and at follow‐up after readaptation gravitational loading on Earth. Regression lines (dark blue) with 95% confidence intervals (light blue).

As there is a close connection between bone and muscle health, and both bone and muscle properties decline with spaceflight, we investigated whether the relationship between spinal muscle and vertebral trabecular bone was altered due to spaceflight. Before flight, global Tb.vBMD was positively correlated with both paraspinal muscle density (*R* ^2^ = 0.38, *p* = 0.0087) and CSA (*R* ^2^ = 0.26, *p* = 0.035) (Fig. 5). Immediately following spaceflight, global Tb.vBMD remained significantly correlated with muscle CSA (*R* ^2^ = 0.32, *p* = 0.017) but not muscle density (*R* ^2^ = 0.11, *p* = 0.19) (Fig. 5). Following a year of readaptation to gravitational loading, the muscle/bone density relationships were opposite the postflight time point, with global Tb.vBMD positively correlated with muscle density (*R* ^2^ = 0.4, *p* = 0.011) but not total muscle CSA (*R* ^2^ = 0.17, *p* = 0.12) (Fig. 5). Finally, we sought to determine whether astronauts who had greater losses in bone mass also experienced greater muscle loss. The change in Tb.vBMD was not correlated with changes in muscle density or CSA either during spaceflight or during readaptation (*p* > 0.1 for all). We also considered whether astronauts with greater bone and muscle density tended to lose more bone during spaceflight. However, preflight bone density was not associated with percentage change in bone density following flight (*R* ^2^ = 0.134, *p* = 0.609) nor percentage change in bone density following readaptation (*R* ^2^ = 0.0289, *p* = 0.91). Similarly, no relationships between baseline muscle density and cross‐sectional area were detected with changes in muscle measures during spaceflight or recovery.

## Discussion

While spaceflight‐induced deficits in vertebral bone density and paraspinal musculature are well established,^(^ ^1^, ^9^, ^13^ ^)^ the relationships among these musculoskeletal changes are less well understood. Here we demonstrated that astronauts lost trabecular bone in the lumbar spine while in space. Yet we found a high degree of individual variability in crewmembers upon their return to Earth, as some astronauts continued to lose trabecular bone, while others recovered to preflight levels. Additionally, our results indicated that changes in vertebral trabecular bone density during spaceflight might not be homogeneous, with greater losses appearing to occur in the superior versus the inferior region of the vertebral body. Further, whereas paraspinal muscle loss occurs during spaceflight, astronauts recovered muscle cross‐sectional area upon their return. Changes in the association between vertebral bone and paraspinal muscle density also suggest a decoupling of the bone–muscle relationship, perhaps due to increased fatty infiltration of musculoskeletal tissues during and following spaceflight. Thus, our results may provide insight into potential mechanisms for increased rates of low back pain, vertebral fracture, and disc herniation following spaceflight.

Consistent with previous studies,^(^ ^1^, ^9^, ^18^ ^)^ we found that spaceflight results in the variable loss of trabecular bone in the spine with limited (and variable) readaptation upon return to Earth. We demonstrated a slightly greater loss in bone density in the superior region than in the overall measure of vertebral vBMD. While this finding needs to be validated in a larger population, it suggests the superior region of vertebrae is more responsive to disuse than other regions of the bone, and changes to the superior region may be detectable before changes to the global measure. Extending these observations, we investigated whether the vertebral bone loss following spaceflight was heterogeneous, hypothesizing that bone changes would be greatest in the regions closest to the endplates, that is, in the superior and inferior regions of the vertebral body. In this population, the superior region of the vertebral body had a marginally greater rate of bone loss during spaceflight than the transverse or inferior regions. Yet the inferior vertebral region was highly conserved, without any significant differences either following spaceflight or 1 year of readaptation. This asymmetric rate of bone loss in regions adjacent to the vertebral endplates may alter disc loading and contribute to postflight endplate pathologies in astronauts, such as disc herniation, decreased range of motion, localized back pain, and spine stiffness.^(^ ^9^, ^13^ ^)^ Reasons for the conservation of the inferior region are unclear. Additionally, the transverse region demonstrated bone loss like that of the superior region during spaceflight but, unlike the superior region, did not regain preflight levels of density after a year of readaptation to gravity. Further studies with increased sample size are needed to confirm these observations, as the sample power in this study was insufficient to provide definitive evidence of higher regions of bone density conservation than others. Nonetheless, this spatial heterogeneity in bone loss may lead to decrements in vertebral strength that are disproportionate to the deficits in vertebral bone density.^(^ ^7^ ^)^

Previous work demonstrated a decline in the ratio of superior to transverse Tb.BMD in lumbar vertebrae with increasing age,^(^ ^7^ ^)^ and our findings of higher rates of bone loss in the superior versus transverse and inferior regions within the vertebral body are consistent with the notion of spaceflight being a model for accelerated aging. Longer‐duration missions are likely needed to see additional manifestations of differences in intravertebral trabecular BMD. Indeed, Gabel et al. examined HR‐pQCT scans of the distal radius and tibia and noted that astronauts on a more extended mission (>7 months) experienced substantially greater bone density and bone strength declines after spaceflight than those crewmembers on shorter‐duration (<6 months) missions.^(^ ^18^ ^)^ Additionally, Gabel et al. found that individuals on long‐duration missions had less robust readaptation of bone density following their return to Earth. Thus, the changes due to roughly 6 months of space travel may be insufficient to characterize regional changes in vertebral trabecular bone density.

Of particular interest, paraspinal muscle density and cross‐sectional area did not follow the same pattern of readaptation in astronauts after their mission. Muscle CSA improved during the 1‐year readaptation period, but muscle density did not. The decline in muscle density suggests fat infiltration in the paraspinal muscles, which is associated with muscle degeneration and dysfunction.^(^ ^19^ ^)^ The paraspinal muscles are important spinal stabilizers, specifically intersegmental stability, and contribute 60% to 80% of the active stiffness imparted on the lumbar spine.^(^ ^20^ ^)^ Spaceflight‐induced paraspinal muscle deconditioning may influence astronauts' balance, mobility, and injury prevention upon their return to Earth. Thus, the loss of muscle density, rather than the loss of muscle CSA, appears to have a greater impact on spinal health than decrements in muscle size and highlights the importance of future studies of fat infiltration in the muscles of astronauts on long‐duration missions.

The concomitant loss of bone and muscle density during spaceflight may be related to fatty infiltration of both tissues. In our study, muscle density and bone density remained positively correlated during the follow‐up time, suggesting, in some astronauts, that these two tissue types continued to degrade together after the mission. Nine of the 17 crewmembers in this study provided self‐reported in‐flight exercise logs. These nine crewmembers averaged 98 ± 41 min/week on the treadmill, 89 ± 52 min/week on the cycle ergometer, and 4.5 ± 1.2 sessions/week of resistance exercises (Interim Resistive Exercise Device or Schwinn Exercise Device) with loads up to 300 lb of force for various exercises.^(^ ^9^ ^)^ In these nine crewmembers Burkhart et al. observed a protective effect of in‐flight resistance exercise against loss of muscle CSA; however, none of the exercises correlated with muscle density.^(^ ^9^ ^)^ These exercise regiments may explain some of the variation in the muscle density, cross‐sectional area, and bone density measures among astronauts in this study. In terrestrial patients, fatty infiltration of the paraspinal muscles is negatively correlated with vertebral BMD^(^ ^21^ ^)^ and is an independent predictor of fragility fractures.^(^ ^21^, ^22^ ^)^ Further, fatty infiltration of muscle has been associated with increasing vertebral bone marrow fat,^(^ ^23^ ^)^ which in turn is associated with a loss of trabeculae.^(^ ^24^, ^25^ ^)^ Importantly, for astronauts returning to Earth, muscle and bone density loss, fatty infiltration, and fragility fractures are of concern for long‐term health and mission readiness. Potential therapeutics that target both muscle and bone, such as activin A decoy receptors, could prevent or even restore both muscle and bone density and should be investigated further.^(^ ^26^ ^)^

The association between paraspinal muscle density and vertebral trabecular bone density weakens in the immediate postflight period, reflecting a high degree of interastronaut variability in the recovery of trabecular BMD. This change in the association between muscle and bone measures was driven by the variability of trabecular bone mineral density at the 1‐year follow‐up, as some astronauts continued to lose trabecular BMD (Tb.vBMD), while others recovered to preflight levels. Yet we did not find an association between change in loss in bone density and muscle density in this population. Thus, whether or not astronauts who lose more muscle also lose more BMD requires further investigation with a larger sample size. Future work is needed to determine the driver of the variation in Tb.vBMD during the readaptation period, as none of the other measures in this study (flight duration, BMI, age) were significantly associated.

Our study has several limitations. As with all spaceflight research, this work had a limited sample size and statistical power. As space travel becomes more accessible, future studies may not only have a larger sample population but also leverage a more diverse data set to investigate sex‐, race‐, and age‐based differences. Spaceflight‐induced bone loss is suspected to be nonlinear^(^ ^27^ ^)^ and progressive.^(^ ^28^ ^)^ Bone density during the readaptation period may likewise occur at a nonlinear rate, and additional measurement time points would help to elucidate bone loss and recovery rates. There is still an unmet need for an in‐flight monitoring device for bone and muscle health, which could be valuable for assessing musculoskeletal health during long‐duration missions and offering personalized interventions and countermeasures. Our study was also limited to the lumbar vertebrae and could be improved with additional spinal sites to determine whether certain regions of the spine are at greater risk of bone and muscle loss.^(^ ^15^, ^16^ ^)^ Functional assessments of the trunk muscles would also provide insight into the changes in muscle strength, rather than size and density alone.

In conclusion, in this prospective longitudinal study of astronauts and cosmonauts, we found that spaceflight had a longer lasting negative effect on vertebral trabecular bone density than on the size of the paraspinal muscles, which appears to recover with reloading during the first year. These decrements in spinal muscle and bone density may help explain the elevated risk of lumbar disc herniation in astronauts upon their return to Earth. Small sample size and biological variation notwithstanding, our findings also suggest regional variation in spaceflight‐induced bone loss in the vertebrae, with greater losses in the superior region that may contribute to endplate irregularities. Knowledge about these potential changes may better inform our understanding of postflight lower back pain that arises from endplate pathology or disc degeneration. Collectively, the results from our study provide potential insight into the mechanisms of lower back pain in the astronaut population and may aid in the development of novel countermeasures for future spaceflight missions.

## Author Contributions

JC, FJ, KB, HB, and BA collected and analyzed the data. JC and FJ performed the statistical analyses. JC, FJ, and MLB wrote the manuscript with input from all the authors. FJ and MLB conceived the study and oversaw the study conduct.

## Disclosures

The authors have nothing to disclose. The views expressed in this paper are those of the authors and do not necessarily reflect the official positions or policies of the National Aeronautics and Space Administration or the US government.

### Peer Review

The peer review history for this article is available at https://www.webofscience.com/api/gateway/wos/peer-review/10.1002/jbm4.10810.

## Data Availability

The data that support the findings of this study are available from the corresponding author upon reasonable request.
